# Impacts of intestinal microbiota metabolite trimethylamine N-oxide on cardiovascular disease: a bibliometric analysis

**DOI:** 10.3389/fmicb.2024.1491731

**Published:** 2025-01-06

**Authors:** Xiaohui Leng, Xiunan Wei, Jun Wang, Xiaodong Yao, Miaomiao Zhang, Dajuan Sun, Junwei Liang, Lili Chi, Yan Cheng

**Affiliations:** ^1^First College of Clinical Medicine, Shandong University of Traditional Chinese Medicine, Jinan, China; ^2^Yantai Yuhuangding Hospital, Yantai, China; ^3^Affiliated Hospital of Shandong University of Traditional Chinese Medicine, Jinan, China

**Keywords:** bibliometric analysis, trimethylamine N-oxide, cardiovascular disease, microbiota metabolite, gut microbiota, atherosclerosis, inflammation

## Abstract

**Background:**

Trimethylamine N-oxide (TMAO), a metabolite dependent on intestinal microbiota, is closely related to the emergence, progression, and prognosis of cardiovascular disease (CVD), and has received increasing attention in recent years.

**Objective:**

The current research hotspots and future development trends in TMAO and CVD field are found through bibliometrics analysis, which provides reference for further study.

**Methods:**

The bibliometrics tools VOSviewer and CiteSpace were used to analyze the publications from the Web of Science Core Collection (WOSCC) database. The articles published from 2004 to 2024 about the relationship between TMAO and CVD were retrieved. Bibliometric analysis includes annual publications, countries/regions, institutions, authors and co-cited authors, journals and cited-journals, references and keywords.

**Results:**

After searching and screening, 1,466 publications were included for subsequent bibliometric analysis. Since 2014, the number of publications exposing the relationship between TMAO and CVD has increased rapidly, as has the frequency of citations. China, USA and Italy are the countries that publish the most relevant research. Cleveland Clinic is the leading institution in this field. Stanley L Hazen, Zeneng Wang and W H Wilson Tang are the most prolific authors in this field, and the latter two have the closest academic cooperation. *American Journal of Clinical Nutrition* and *Journal of the American Heart Association* are influential journals that publish research in this field. “*Gut Microbial Metabolite TMAO Enhances Platelet Hyperreactivity and Thrombosis Risk*” is the most frequently cited article. Keyword analysis shows that gut microbiota, metabolism, phosphatidylcholine and atherosclerosis (AS) are the hotspots in this field.

**Conclusion:**

This study summarizes the research situation of TMAO and CVD in the past 20 years, focusing on the effect of TMAO on pathogenesis of AS, predictive value of TMAO on CVD risk, and dietary and drug intervention for TMAO. Probiotics and natural products may be the research focus of preventing and treating CVD by intervening TMAO in the future.

## 1 Introduction

Cardiovascular disease (CVD) is the main cause of death of non-communicable diseases worldwide, accounting for about 30% of all deaths (Mahmood et al., 2014). As the population grows and aging intensifies, the count of deaths and disabled individuals linked to CVD continues to rise (Roth et al., 2020). Research indicates that between 1990 and 2015, the global death toll due to CVD rose from 12.59 to 17.92 million (Al-Mallah et al., 2018). Another survey has shown that from 1990 to 2019, the annual death toll of global premature CVD (15–49 years old) increased by 25%, which indicates more serious health risks and medical burden (Lababidi et al., 2023). Although recent research shows that the global age-standardized CVD mortality rate has declined, this situation only appears in countries with high per capita income. In contrast, the mortality rate in most underdeveloped areas remains at the original level (Vogel et al., 2021). Ischemic heart disease (IHD), stroke, and heart failure (HF) rank as the top three reasons for deaths linked to CVD (Joseph et al., 2017). Indeed, managing the changeable risk elements can significantly lower the likelihood of disability and premature death in the CVD population (Roth et al., 2020). Therefore, the exploration and control of risk factors has always been a research hotspot in CVD field.

Trimethylamine N-oxide (TMAO) is an intestinal microbiota-reliant metabolite in plasma. The gut microbiota breaks down choline, l-carnitine, and phosphatidylcholine from the diet to form trimethylamine (TMA), which is subsequently taken into the bloodstream and converted into TMAO by flavin-containing monooxygenase 3 (FMO3) in the liver (Chen et al., 2019). In recent years, the influence of TMAO on CVD has been widely reported, and it has been proved as an important medium to participate in the occurrence and development of various CVDs, including hypertension (Zhang W. Q. et al., 2021), HF (Zhang Y. et al., 2021) and atherosclerotic CVD (Canyelles et al., 2023). Meanwhile, many studies show that TMAO is also a powerful prognostic marker, and the increase of its circulation level is closely related to the increased risk of CVD-related adverse events (Guasti et al., 2021; Aleksova et al., 2024; Chen et al., 2022). Although the mechanism of TMAO leading to the occurrence and development of CVD has not been fully clarified, existing studies have shown that TMAO plays an important role in promoting the key pathological links of CVD such as thrombosis (Zhu et al., 2016), atherosclerosis (AS) (Koeth et al., 2013) and endothelial dysfunction (Querio et al., 2023), so TMAO is likely to be a promising intervention target to control the progress of CVD.

The purpose of bibliometrics is to depict or illustrate the connections among different details in published works by statistically analyzing these publications and their associated metadata (Ninkov et al., 2022). This method can describe the general development track of a field of interest and help to find research hotspots and high-impact articles/institutions/authors in this field. Currently, some scholars have conducted bibliometric analyses on the relationship between the gut microbiota, its metabolic products, and the occurrence and development of CVD. Mu et al. (2022) identified TMAO as a hot topic through a bibliometric analysis of the gut microbiota and HF, Wang Y. et al. (2022) found that choline, the precursor of TMAO, was a key focus of attention through a bibliometric analysis of the gut microbiota and atherosclerosis. Han et al. (2023) identified TMAO as one of the clusters in the keyword co-occurrence analysis, with its precursor phosphatidylcholine appearing in the most recent keyword explosions, through a bibliometric analysis of stroke and the gut microbiota. Therefore, TMAO has become a research hotspot in the field of CVD and the gut microbiome in recent years. However, in previous studies, the metabolic product TMAO has only appeared as a minor component and has been explored mainly in the context of a single pathological mechanism or disease, without forming a comprehensive landscape of how TMAO impacts CVD. This research aims to scrutinize the two decades-long literature linking TMAO and CVD, encapsulate the evolution of this domain, identify key areas of research, and forecast upcoming trends, thereby serving as a guide for advancing TMAO and CVD studies.

## 2 Methods

### 2.1 Data sources

Literature retrieval is carried out through the Web of Science Core Collection (WOSCC) database, including Science Citation Index Expand, Social Science Citation Index, Current Chemical Reactions and Index Chemicus. The data used are all from public databases, without ethical approval.

### 2.2 Search strategy

**Figure 1:**
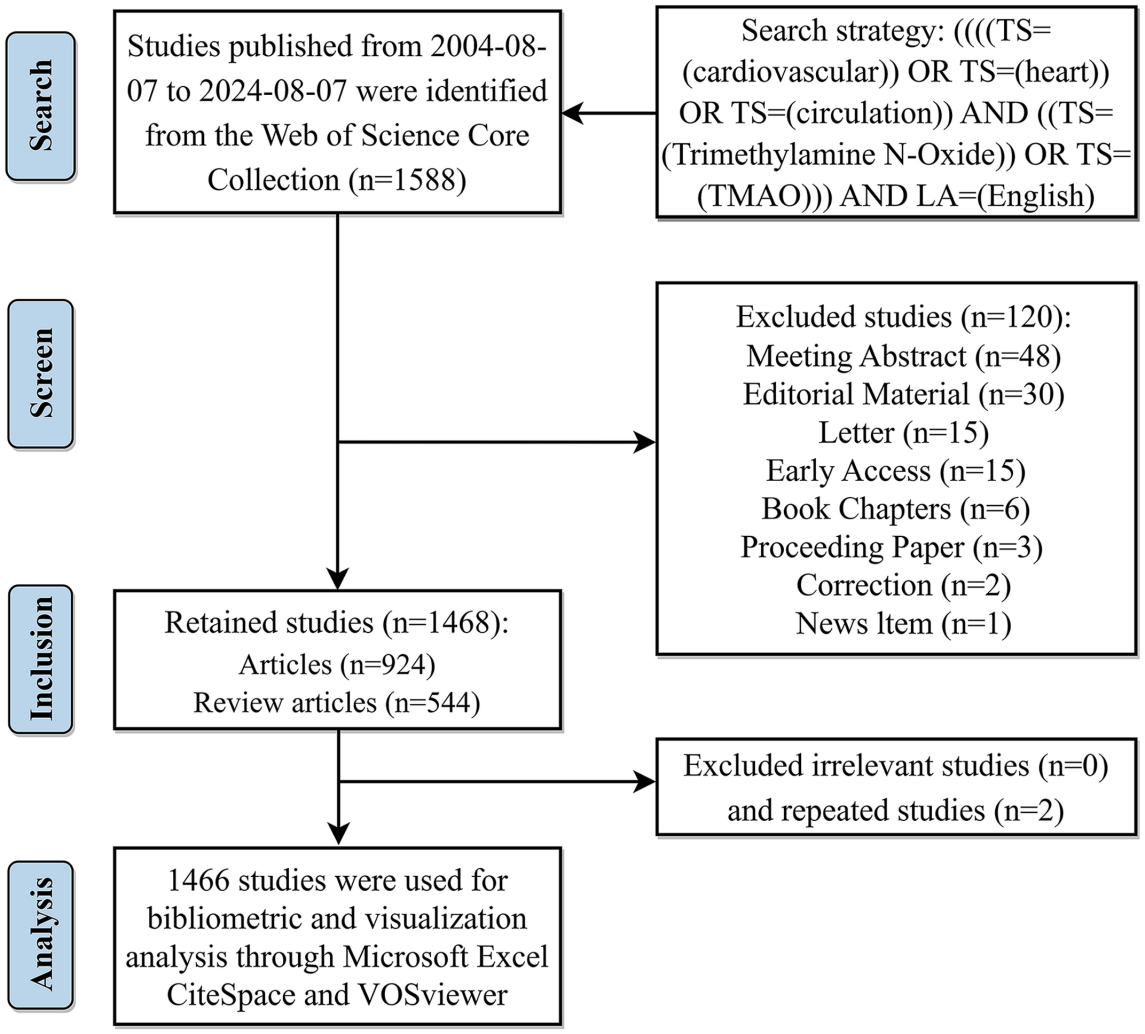
Flowchart of literature screening. Created by figdraw.

The following keywords and search strategies were employed in referring to the studies of Tian et al. (2024) and Mao et al. (2023): [((((TS = (cardiovascular)) OR TS = (heart)) OR TS = (circulation)) AND ((TS = (Trimethylamine N-Oxide)) OR TS = (TMAO))) AND LA = (English)]. In order to avoid data bias caused by daily updating of the database, the research related to TMAO and CVD published in the past 20 years (from August 7, 2004 to August 7, 2024) was retrieved and downloaded within 1 day (August 8, 2024). A total of 1,588 records were obtained, including 10 document types, namely, article, review article, meeting abstract, editorial material, letter, early access, book chapters, proceeding paper, correction and news item, among which the last 8 document types were excluded. Two independent literature researchers manually re-examined the titles and abstracts of 1,468 articles/review articles, so as to eliminate irrelevant and repetitive studies and ensure that they meet the established standards. Finally, 1,466 articles were included for subsequent bibliometric analysis. Figure 1 illustrates the precise procedure.

### 2.3 Data extraction and bibliometric analysis

Export the “Full Record and Cited References” of the selected publication in the format of “Plain Text File.” This data set contains information such as titles, authors, institutions, abstracts, keywords, references, etc. Microsoft Excel 2021 was used for initial data analysis. VOSviewer (version 1.6.20) and CiteSpace (version 5.5.R2) were used for advanced bibliometric analysis and visualization.

Specifically, Microsoft Excel 2021 was used to create a visual picture of the annual publication, citation trends and the publishing trends of various countries/regions. VOSviewer contributed to the analysis and visualization of the countries/regions, authors and co-cited authors, journals and cited-journals and keywords of the publications. The Gross Domestic Product (GDP) of each country is extracted from the World Bank online database,^1^ and the research productivity of different countries is further standardized (Stefanis et al., 2022). While, CiteSpace was applied to analyze and visualize the institutions, cited references, references bursts and keywords bursts.

## 3 Results

### 3.1 Annual publication and citation trend

**Figure 2:**
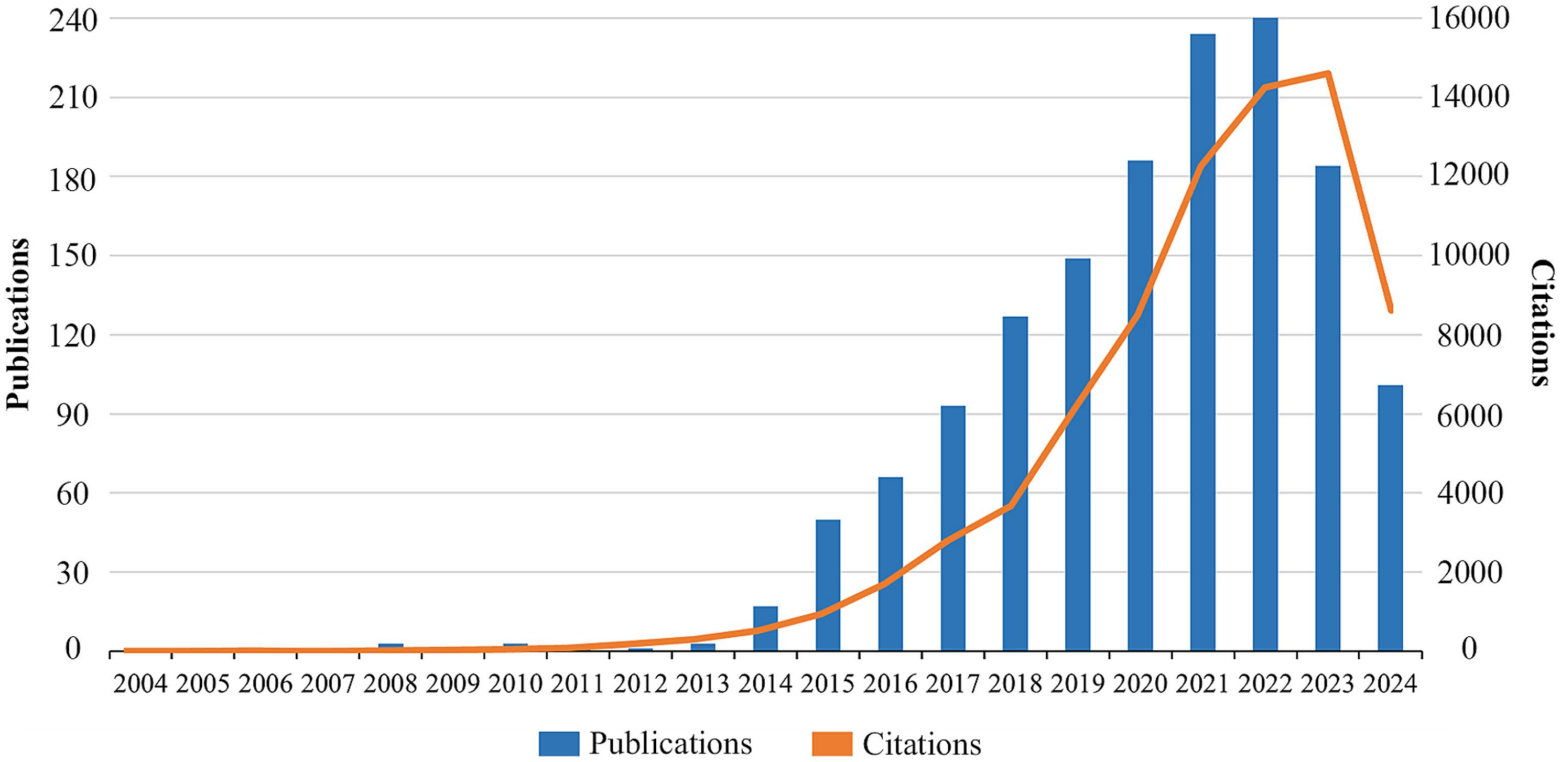
Annual publication and citation trends in the past 20 years.

Figure 2 shows the annual publication and citation trend of TMAO and CVD related literature. In 2005, for the first time, researchers found that the heart tissue of smelt could accumulate TMAO, which was different from the fluctuation of TMAO level in other organs with plasma TMAO (Treberg et al., 2005). In 2008, Lewis et al. (2008) first observed the value of TMAO in the early detection of myocardial injury through the emerging metabonomics tools at that time, thus opening a new research field. Since then, until 2013, the number of related studies has remained in single digits every year, which shows that TMAO and cardiovascular diseases did not attract scholars’ interest and attention at that time. Since 2014, the number of annual publications has exceeded 10, and it has increased at a very rapid rate. By 2022, it has reached the peak of 248 articles, which is accompanied by the rapid increase of citation. In 2023, the number of related publications decreased, which was related to the prevalence of COVID-19 virus and the improvement of the quality requirements of papers in various countries, institutions and publishing houses, which led to the decline of the number of publications in various fields (Mallapaty, 2021). However, the number of citations in this field is still increasing, which shows that the research on TMAO and cardiovascular diseases is still getting more and more attention.

### 3.2 Countries/regions and institutions distribution

**Figure 3:**
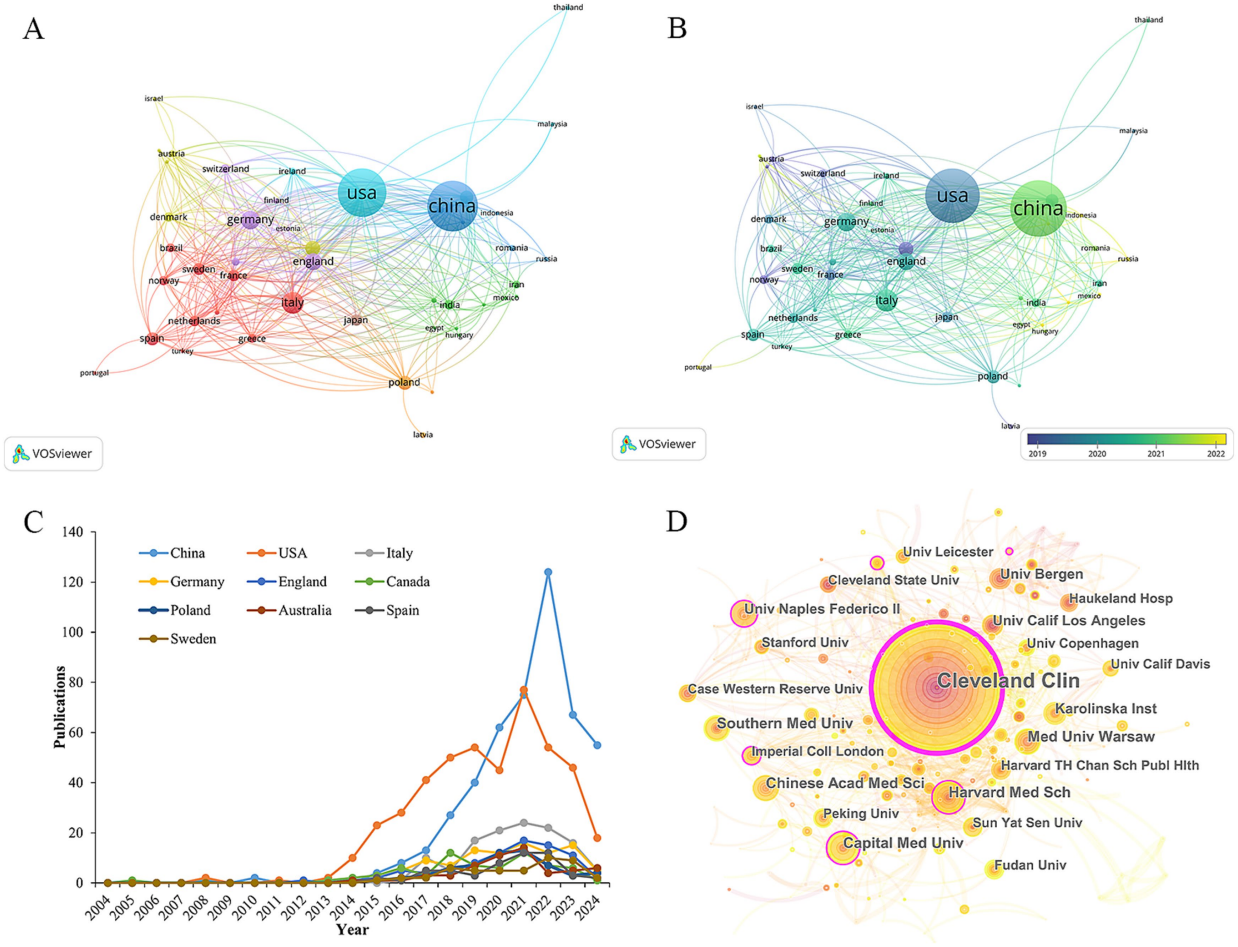
Visualization of countries/regions and institutions. **(A)** Countries/regions cooperation network. **(B)** Countries/regions cooperation network with overlapping time. **(C)** The annual trend of the top 10 countries/regions. **(D)** Institutions distribution and inter-agency cooperation network diagram. The nodes represent institutions, and their size is proportional to the number of publications. The lines between the nodes represent collaborations, and the thicker the line, the closer the collaboration. Nodes with purple edges indicate that they have high centrality and are the key nodes in the network.

The publications on TMAO and CVD cover 79 countries/regions. Table 1 enumerates the top 10 most productive countries/regions at present. China ranks first with 478 publications, followed by USA (451), Italy (122), Germany (96) and other countries. In terms of total citations, average citations and H-index, which reflect the influence of the article, USA ranks first, highlighting the outstanding contributions of American scholars in the research fields related to TMAO and CVD. Considering the influence of GDP, Sweden (9.55) is the most productive country in this field, and its average citation (52.89) is second only to USA (89.69) and Germany (55.25), which shows that although the number of its articles is small, its research has a high influence in this field. In VOSviewer, total link strength (TLS) can reflect the total strength of the co-author links of a given country/institution/author with others (Wu et al., 2024). Through the target TLS and Figure 3A, it can be seen that USA (TLS: 343), Germany (TLS: 190), and England (TLS: 183) have close international cooperation with other countries/regions. In addition, as shown in Figure 3B, compared with the United States (451), China (478), the country with the largest number of articles, the year when China scholars published research on TMAO and cardiovascular diseases is more recently. Referring to Figure 3C, the number of articles published by USA began to grow rapidly in 2014 and reached its current peak in 2021. Although China’s publishing volume only entered a period of rapid growth in 2017, its publishing volume surpassed that of USA for the first time in 2020 and reached its current peak in 2022. This suggests that the research on TMAO and CVD is becoming a new research hotspot among Chinese scholars. It is worth noting that the influence of research in China (H-index: 57) is not as good as that in the United States (H-index: 93), which may be due to the late start of research in this field in China and the relatively weak cooperation with other countries. In addition, countries such as Türkiye, Portugal, Lithuania have also begun to study TMAO and cardiovascular diseases in recent years.

**Table 1:** Top 10 productive countries/regions.

No.	Country/ region	Articles	Articles/trillion GDP	Total citations	Average citation	H-index	TLS
1	China	478	4.68	13,510	28.26	57	131
2	USA	451	2.51	40,448	89.69	93	343
3	Italy	122	6.43	4,226	34.64	29	120
4	Germany	96	2.91	5,304	55.25	35	190
5	England	83	2.91	3,924	47.28	33	183
6	Canada	67	4.40	3,131	46.73	29	101
7	Poland	59	1.26	2,347	39.78	26	54
8	Australia	56	4.30	2,105	37.59	26	81
9	Spain	53	4.35	2,732	51.55	24	60
10	Sweden	47	9.55	2,486	52.89	22	99

The publications of TMAO and CVD come from 322 academic institutions. Table 2 displays the top 10 productive institutions. Cleveland Clinic is far ahead with an absolute advantage of 102 articles, and they first confirmed that TMAO is a metabolite of phosphatidylcholine in the intestinal microbiota through animal experiments, and confirmed its role in promoting atherosclerosis (Wang et al., 2011). Prior to this, the understanding of TMAO was limited to one of the products of plasma metabonomics in patients with CVD. All these show that Cleveland Clinic is a leading unit in the research field of TMAO and CVD in the world. Followed by Harvard Medical School (27), Capital Medical University (26) and Medical University of Warsaw (25). Within CiteSpace, the centrality index may indicate a node’s significance in the network. Should the centrality exceed 0.1, the node is considered crucial (Lv et al., 2023). Referring to the centrality index and the inter-agency cooperation network diagram (Figure 3D), it can be seen that among the top 10 institutions, Cleveland Clinic (0.45), Harvard Medical School (0.13), Capital Medical University (0.11) and The University of Naples Federico II (0.11) is located at the key nodes of the network, which shows that these institutions have made outstanding contributions in the research field of TMAO and CVD, and played a role as a link in international cooperation.

**Table 2:** Top 10 productive institutions.

No.	Institution	Country/region	Articles	Centrality	Year
1	Cleveland Clinic	USA	102	0.45	2013
2	Harvard Medical School	USA	27	0.13	2017
3	Capital Medical University	China	26	0.11	2018
4	Medical University of Warsaw	Poland	25	0.07	2017
5	Southern Medical University	China	23	0.01	2017
6	Chinese Academy of Medical Sciences	China	23	0.04	2019
7	Karolinska Institute	Sweden	21	0.08	2018
8	University of Naples Federico II	Italy	21	0.11	2017
9	University of California, Los Angeles	USA	19	0.01	2015
10	University of Bergen	Norway	19	0.03	2015

### 3.3 Analysis of authors and co-cited authors

**Figure 4:**
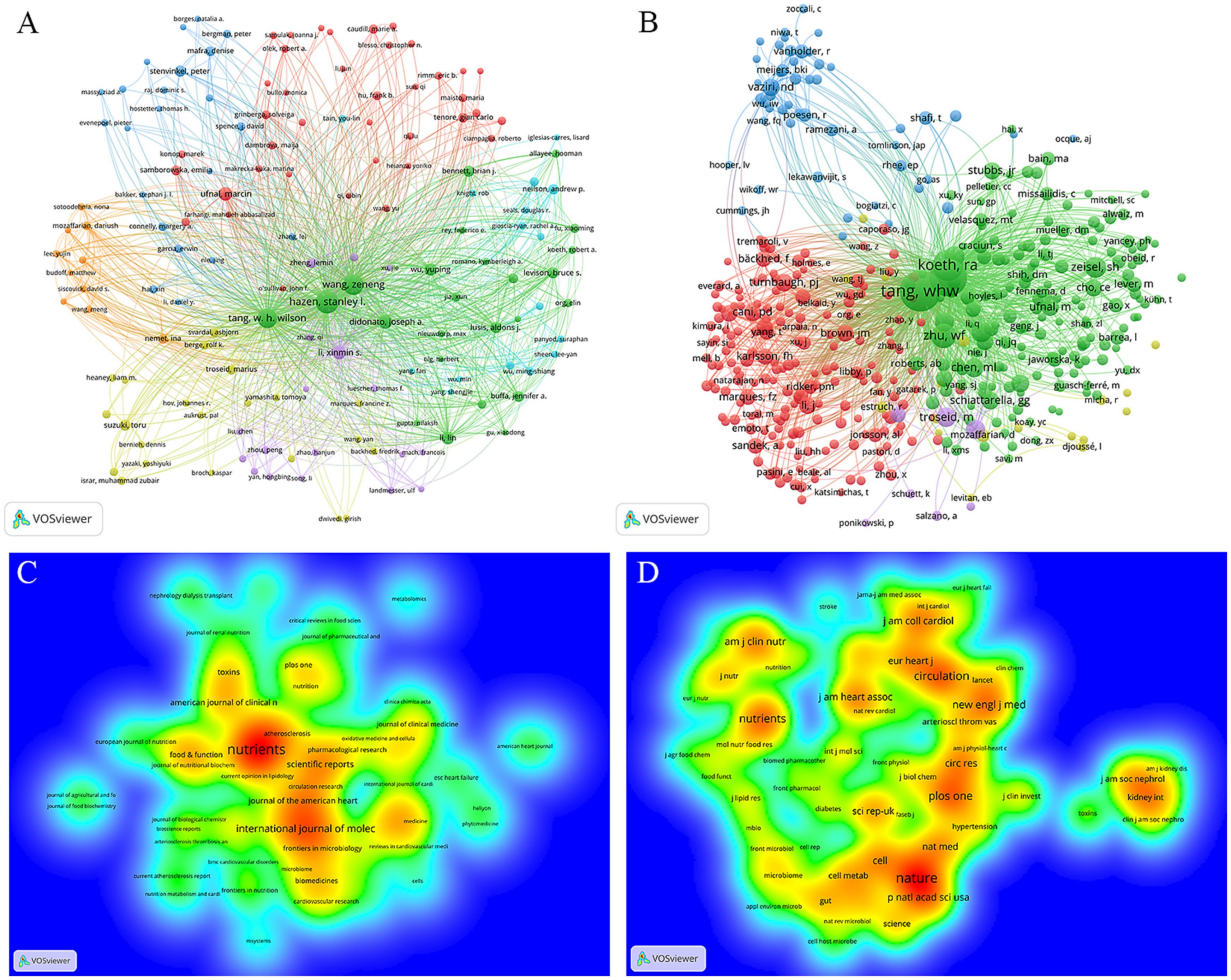
Visualization network of authors and journals. **(A)** Network analysis diagram of authors. **(B)** Network analysis diagram co-cited authors. The nodes represent the authors, and their size is proportional to the number of publications or the frequency of co-citations. The connections between the nodes represent collaborative relationships. **(C)** The density distribution map of journals. **(D)** The density distribution map of cited journals. The color ranges from red to yellow to green to blue, indicating that the density decreases in turn. The more items in the neighborhood of a journal, the higher the number of publications or citations related to the journal, and the closer the color of the point is to red. On the contrary, the fewer items in the neighborhood of a journal, the lower the number of publications or citations related to the journal, and the closer the color of the dots is to blue.

The publications on TMAO and CVD involve 8,135 authors. Bibliographic coupling analysis method in VOSviewer can make use of the cited documents shared between papers to generate a relational network, which can reflect the correlation between projects (Stefanis et al., 2023). The node size reflects the number of the author’s papers, and the proximity between nodes reflects the degree of correlation between the topics studied by the author. The authors with the number of papers ≥5 were visualized by VOSviewer, and 151 authors were included, as depicted in Figure 4A. Table 3 displays the top 10 authors who produced the most articles, of which 7 were from Cleveland Clinic in USA. In addition, the author’s network diagram contains 7 clusters. Stanley L Hazen ranks the highest in the green cluster, and also ranks the highest in the whole author network. His research mainly focuses on the correlation between TMAO and the occurrence, development and prognosis of CVD such as AS and HF (Tang et al., 2024; Wang et al., 2023). Marcin Ufnal is the most published in the red cluster, and he mainly focuses on the intermediary role of TMAO between diet, intestinal microbiota, gut-blood barrier and CVD (Gąsecka et al., 2023; Drapala et al., 2020). Xin Min Si Li (Tang et al., 2024), Toru Suzuki (Israr et al., 2021) and Ina Nemet (Lemaitre et al., 2023) are the most influential authors in the purple, yellow and orange clusters respectively, and they are all concerned about the predictive value of TMAO for HF, AS, acute coronary syndrome and cardiovascular events. Peter Stenvinkel ranks the first in the blue cluster. He mainly studies the influence of diet on TMAO metabolism and its influence on the prognosis of chronic kidney disease (CKD) (Evans et al., 2023; Hernandez et al., 2022). Andrew P Neilson is a key author in the light blue cluster, and TMAO’s aging-related vascular oxidative stress and endothelial dysfunction are its main areas of concern (Brunt et al., 2020; Brunt et al., 2019).

**Table 3:** Top 10 authors.

No.	Author	Country	Institution	Articles	Citations	H-index	TLS
1	Stanley L Hazen	USA	Cleveland Clinic	72	21,701	51	424,344
2	Zeneng Wang	USA	Cleveland Clinic	57	18,612	40	334,254
3	W H Wilson Tang	USA	Cleveland Clinic	51	17,835	40	304,504
4	Joseph A DiDonato	USA	Cleveland Clinic	23	10,916	19	141,363
5	Xinmin S Li	USA	Cleveland Clinic	23	2,659	18	145,827
6	Marcin Ufnal	Poland	Medical University of Warsaw	23	1,167	16	78,215
7	Lin Li	Sweden	Karolinska Institute	20	6,640	17	106,125
8	Yuping Wu	USA	Cleveland Clinic	19	14,449	18	104,903
9	levison, bruce s.	USA	Cleveland Clinic	16	13,428	15	83,563
10	Aldons J Lusis	USA	University of California	16	11,221	15	96,749

Co-cited authors network reflects the academic relevance among authors. The references of TMAO and CVD publications cover a total of 42,859 authors. Through the visual analysis of the authors with citation times ≥30 by VOSviewer, a total of 422 co-cited authors met this standard, as shown in Figure 4B. It is worth noting that Zeneng Wang and W H Wilson Tang from Cleveland Clinic are the two most co-cited authors, indicating that they have very strong academic cooperation and high academic influence in the research fields related to TMAO and CVD. In addition, Zeneng Wang and W H Wilson Tang also have the highest TLS in the co-cited authors network, indicating that they have also made outstanding contributions in collaboration with other authors/institutions.

### 3.4 Analysis of journals and cited-journals

The publications about TMAO and CVD are published in 544 journals. Through VOSviewer, a visual analysis was made on the journals with the number of published documents ≥5. A total of 69 journals met this standard, as shown in Figure 4C. Table 4 displays the top 10 journals in terms of published articles and cited amount. Among them, *Nutrients* topped the list with 70 articles. Among the top 10 journals, 6 journals are published in Switzerland, and the rest are published in England and USA. The impact factor (IF) of *American Journal of Clinical Nutrition*, *Journal of the American Heart Association* and *Food and Function* exceeds 5 points. It is worth noting that although *Journal of the American Heart Association* has published <1/3 of *Nutrients*, its citations are almost the same as *Nutrients*, which indicates that *Journal of the American Heart Association* has a high academic influence in TMAO and CVD research.

**Table 4:** Top 10 journals with the most publications and citations.

Journals	IF (2023)	Country	Articles	Citations	No.	Cited-journals	IF (2023)	Country	Citations
*Nutrients*	4.8	Switzerland	70	2,812	1	*Nature*	50.5	England	3,182
*International Journal of Molecular Sciences*	4.9	Switzerland	38	770	2	*Circulation*	35.5	USA	2,489
*Scientific Reports*	3.8	England	29	1773	3	*Plos one*	2.9	USA	2,346
*American Journal of Clinical Nutrition*	6.5	USA	24	1,039	4	*Nutrients*	4.8	Switzerland	2,330
*Frontiers in Cardiovascular Medicine*	2.8	Switzerland	22	258	5	*New England Journal Of Medicine*	96.2	USA	1967
*Journal of the American Heart Association*	5.0	USA	22	2,324	6	*American Journal of Clinical Nutrition*	6.5	USA	1887
*Toxins*	3.9	Switzerland	22	1,314	7	*Journal of the American Heart Association*	5.0	USA	1784
*Food and Function*	5.1	England	17	403	8	*Circulation Research*	16.5	USA	1762
*Frontiers in Pharmacology*	4.4	Switzerland	17	708	9	*Journal of the American College of Cardiology*	21.7	USA	1751
*Metabolites*	3.4	Switzerland	16	178	10	*Proceedings of the National Academy of Sciences of the United States of America*	9.4	USA	1722

The references about TMAO and CVD publications come from 6,410 journals. Through VOSviewer, a visual analysis was made on the journals cited ≥300 times. A total of 70 journals met this standard, as shown in Figure 4D. The IF of the cited journals was generally high, and 8 of them had IF exceeding 5 points. *Nature*, an internationally authoritative comprehensive journal, and *Circulation*, a top magazine in cardiovascular field, rank in the top two with 3,182 and 2,489 citations, respectively. It is worth noting that the *American Journal of Clinical Nutrition* and *Journal of the American Heart Association*, not only ranks in the top 10 publications, but also ranks in the top 10 citations, which shows that these two journals are authoritative in the research field of TMAO and CVD.

### 3.5 Cited references analysis and references bursts

**Figure 5:**
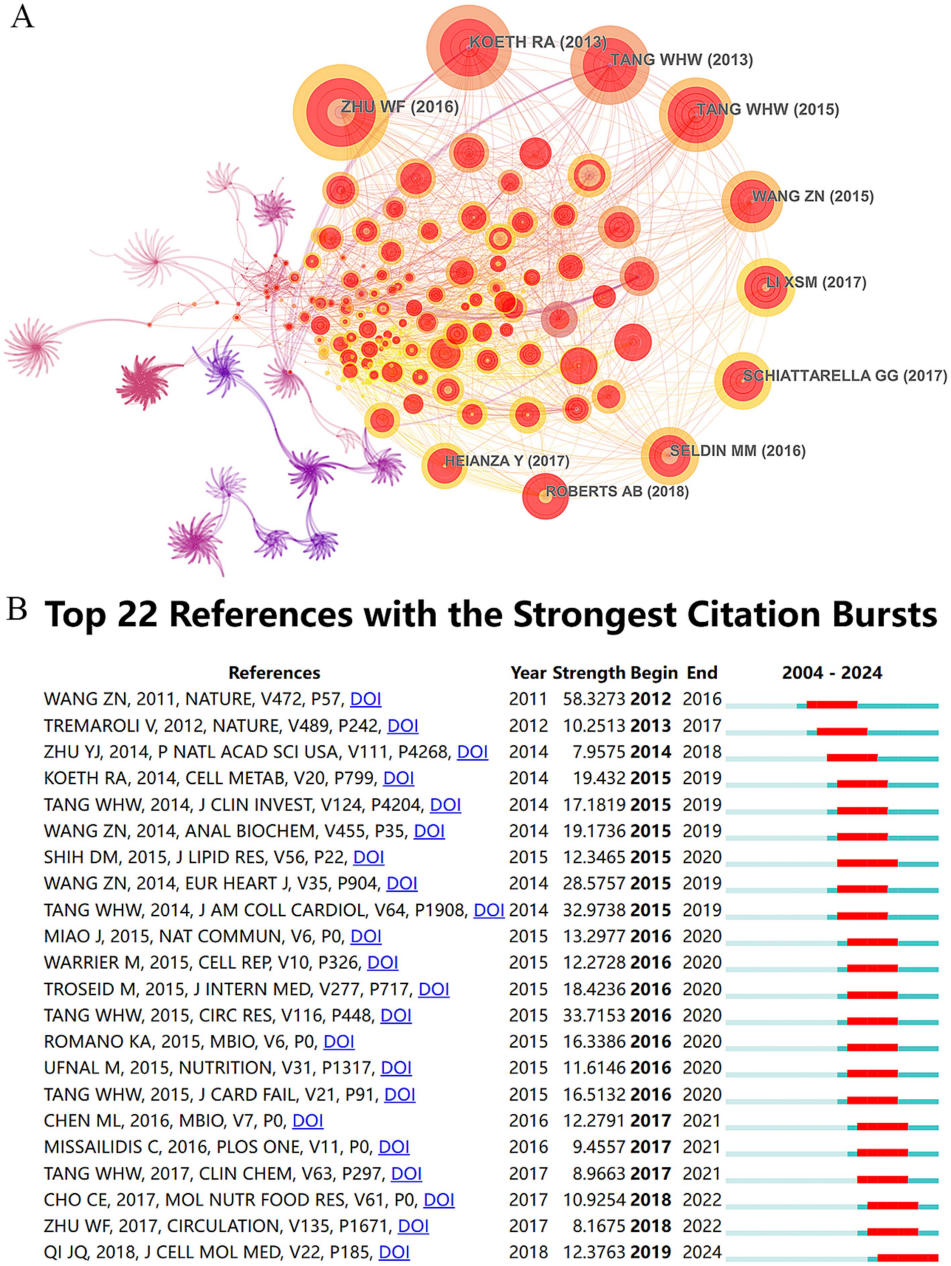
Analytical atlas of cited references and references bursts. **(A)** Network map of cited references. The nodes symbolize the citations cited, with their dimensions corresponding to the frequency of their citations. The interconnections among the nodes symbolize cooperative interactions. **(B)** Top 22 references with the strongest citation bursts in TMAO and CVD.

A total of 62,268 references were cited in the publications on TMAO and CVD, which were visualized by CiteSpace, as shown in Figure 5A. The top 10 cited references are shown in Table 5. Among them, “*Gut Microbial Metabolite TMAO Enhances Platelet Hyperreactivity and Thrombosis Risk*” published in the top international journal *Cell* in 2016 ranked first with 286 frequencies, which is the most frequently cited article so far. This study found that TMAO can predict the risk of myocardial infarction and stroke, and revealed that intestinal microflora can trigger platelet hyperreactivity and thrombosis by producing TMAO, thus providing evidence for TMAO as a risk factor for thrombosis (Zhu et al., 2016). Furthermore, within the top 10 referenced studies, five concentrate on TMAO’s effect on cardiovascular adverse event risks, four on its influence on thrombosis and AS, and one delves into TMAO’s molecular process causing vasculitis.

**Table 5:** Top 10 cited references.

No.	Reference	Journal	Author	Frequency	Year
1	Gut microbial metabolite TMAO enhances platelet hyperreactivity and thrombosis risk	*Cell*	Weifei Zhu	286	2016
2	Intestinal microbiota metabolism of L-carnitine, a nutrient in red meat, promotes atherosclerosis	*Nature Medicine*	Robert A Koeth	252	2013
3	Intestinal microbial metabolism of phosphatidylcholine and cardiovascular risk	*New England Journal Of Medicine*	W H Wilson Tang	234	2013
4	Gut microbiota-dependent trimethylamine N-oxide (TMAO) pathway contributes to both development of renal insufficiency and mortality risk in chronic kidney disease	*Circulation Research*	W H Wilson Tang	221	2015
5	Non-lethal inhibition of gut microbial trimethylamine production for the treatment of atherosclerosis	*Cell*	Zeneng Wang	183	2015
6	Gut microbiota-dependent trimethylamine N-oxide in acute coronary syndromes: a prognostic marker for incident cardiovascular events beyond traditional risk factors	*European Heart Journal*	Xinmin S Li	175	2017
7	Trimethylamine N-oxide promotes vascular inflammation through signaling of mitogen-activated protein kinase and nuclear factor-κB	*Journal of the American Heart Association*	Marcus M Seldin	171	2016
8	Gut microbe-generated metabolite trimethylamine-N-oxide as cardiovascular risk biomarker: a systematic review and dose–response meta-analysis	*European Heart Journal*	Gabriele Giacomo Schiattarella	171	2017
9	Development of a gut microbe-targeted nonlethal therapeutic to inhibit thrombosis potential	*Nature Medicine*	Adam B Roberts	139	2018
10	Gut microbiota metabolites and risk of major adverse cardiovascular disease events and death: a systematic review and meta-analysis of prospective studies	*Journal of the American Heart Association*	Yoriko Heianza	138	2017

In order to analyze the citation trend of highly cited references in recent 20 years, the minimum duration parameter of CiteSpace was set to 5 years, and the references bursts were analyzed, as shown in Figure 5B. The results of references bursts show that a total of 22 references meet the screening conditions, indicating that these references have been continuously cited for more than 5 years in the past 20 years. The specific literature information is shown in Supplementary Table 1. Generally speaking, there are three articles outlining the influence of TMAO on CVD, five articles focusing on the dietary source and metabolism of TMAO, five articles focusing on the influence of TMAO on AS, three articles focusing on the relationship between TMAO and the prognosis of HF, and three articles focusing on the influence of TMAO on cardiovascular adverse events in patients with nephropathy and diabetes. The other three articles focus on the detection method of TMAO, the effect of TMAO on cholesterol and thrombosis. Among them, the influence of “*Gut flora metabolism of phosphatidylcholine promotes cardiovascular disease*” published in the top international journal *Nature* in 2011 still ranks first with a strength of 58.3273. Through large-scale clinical cohort and animal experiments, this study confirmed that TMAO and its dietary precursors, phosphatidylcholine, choline and betaine, can increase the risk of AS by up-regulating the macrophage clearance receptor and the formation of macrophage foam cells related to AS, and this process is dependent on intestinal microflora (Wang et al., 2011). “*Gut flora metabolism of phosphatidylcholine promotes cardiovascular disease*” and “*Gut Microbial Metabolite TMAO Enhances Platelet Hyperreactivity and Thrombosis Risk*,”the two most influential articles, focuses on AS and its related cardiovascular events, which shows that this is the research hotspot of TMAO in CVD field. Significantly, the *Mbio* publication by Chinese academics, titled “*Resveratrol Attenuates Trimethylamine-N-Oxide (TMAO)-Induced Atherosclerosis by Regulating TMAO Synthesis and Bile Acid Metabolism via Remodeling of the Gut Microbiota*” stands as the sole piece among these 22 scholarly works to concentrate on the drug control of TMAO. This study found that resveratrol, a natural product, has prebiotic-like effects, which can regulate TMAO synthesis and bile acid metabolism by remodeling intestinal microflora, thus alleviating TMAO-induced AS (Chen et al., 2016). This provides a new insight for TMAO’s drug intervention, and suggests that intestinal microflora may be an important target for drug or diet intervention in CVD.

### 3.6 Keywords analysis and bursts

**Figure 6:**
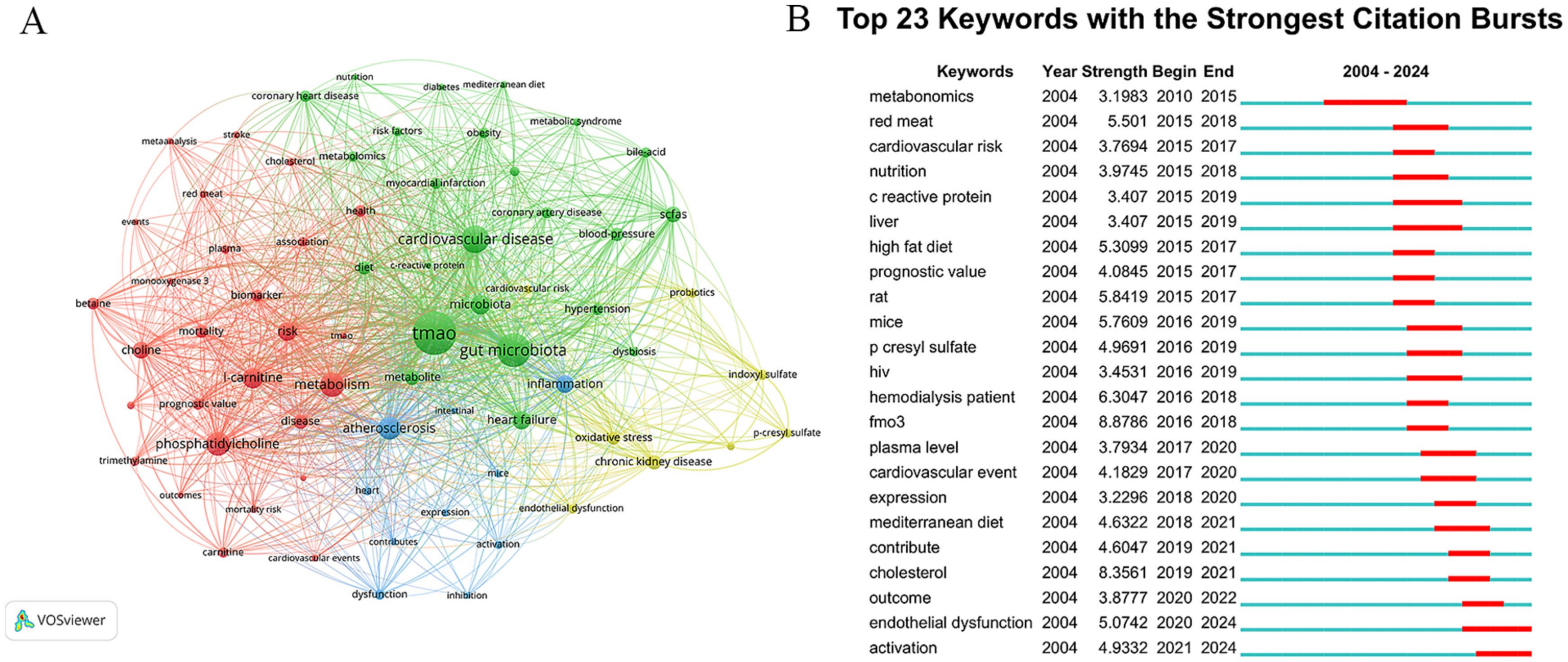
Keywords analysis. **(A)** Clustering network diagram of keywords. The nodes symbolize keywords, with their dimensions correlating to their occurrence rate. The presence of lines signifies the simultaneous emergence of keywords, while denser lines suggest a greater rate of their simultaneous occurrence. Different colors represent different clusters. **(B)** Top 23 keywords with the strongest citation bursts in TMAO and CVD.

There are 4,635 keywords in the publications about TMAO and CVD. The keywords that appear more than 30 times are visualized by VOSviewer, and 69 keywords meet this standard. The clustering network was shown in Figure 6A. The top 20 keywords are shown in Table 6. In addition to TMAO and CVD, the keywords with higher frequency in this study include gut microbiota (697), metabolism (380), phosphatidylcholine (358), AS (327), etc. The list encompasses three illnesses: heart failure, hypertension, chronic kidney disease (CKD), and three pathological conditions: AS, inflammation, and oxidative stress, indicating that they are the focus of research in this field. Keywords clustering can be divided into four clusters, and different colors correspond to different cluster membership relationships, as shown in Table 7. Among them, the red cluster focuses on the dietary metabolism of TMAO and its relationship with the prognosis of the disease, and the key terms include TMAO, trimethylamine, red meat, phosphatidylcholine, l-carnitine, choline, cardiovascular events, mortality, mortality risk, outcomes and prognostic value. The green cluster focuses on the association between TMAO and different CVD risk and metabolites of other intestinal microflora, among which the key terms include TMAO, cardiovascular disease, coronary artery disease, coronary heart disease, heart failure, hypertension, myocardial infarction, risk factors, SCFAs and bile-acid. The blue cluster has some terms such as heart, atherosclerosis, dysfuction, infection, inhibition, and mice, which indicates that atherosclerosis and its mechanism are the hot spots in the experimental research field of TMAO and CVD. The yellow cluster contains cardiovascular risk, oxidative stress, endocrine dysfunction and probes, which suggests that mechanism research and probiotics are also hot topics in this field.

**Table 6:** Top 20 keywords.

No.	Keyword	frequency	TLS	No.	Keyword	frequency	TLS
1	TMAO	1,112	6,578	11	inflammation	210	1,408
2	gut microbiota	697	4,439	12	choline	192	1,379
3	cardiovascular disease	492	3,273	13	SCFAs	167	1,160
4	metabolism	380	2,532	14	disease	139	871
5	phosphatidylcholine	358	2,462	15	chronic kidney disease	136	843
6	atherosclerosis	327	2,344	16	diet	121	901
7	l-carnitine	262	1873	17	oxidative stress	120	676
8	microbiota	246	1,645	18	blood-pressure	117	799
9	risk	237	1,605	19	mortality	108	754
10	heart failure	218	1,442	20	hypertension	97	723

**Table 7:** Keywords grouping by VOSviewer clusters.

Cluster identification	Keywords
Red	association, betaine, biomarker, cardiovascular events, carnitine, cholesterol, choline, containing monooxygenase 3, disease, events, health, l-carnitine, meta-analysis, metabolism, microbial-metabolism, monooxygenase 3, mortality, mortality risk, outcomes, phosphatidylcholine, plasma, prognostic value, red meat, risk, stroke, tmao, trimethylamine
Green	tmao, scfas, bile-acid, blood-pressure, c-reactive protein, cardiovascular disease, coronary artery disease, coronary heart disease, diabetes, diet, dysbiosis, gut microbiota, heart failure, hypertension, insulin-resistance, mediterranean diet, metabolic syndrome, metabolite, metabolomics, microbiota, myocardial infarction, nutrition, obesity, risk factors
Blue	activation, atherosclerosis, contributes, dysfunction, expression, heart, inflammation, inhibition, intestinal, mice
Yellow	cardiovascular risk, chronic kidney disease, endothelial dysfunction, indoxyl sulfate, oxidative stress, p-cresyl sulfate, probiotics, uremic toxins

In addition, we set the minimum duration parameter of CiteSpace to 3 years, and made burst analysis of keywords, as shown in Figure 6B. The results of Keywords bursts show that 23 keywords meet the screening criteria, indicating that these keywords have been research hotspots for at least three consecutive years in the past 20 years. The year 2015 marks a significant juncture. Before 2015, only the word metabonomics met the screening criteria. Metabonomics detection for plasma is an important quantitative means of TMAO. At present, the developed detection methods of TMAO mainly include colorimetry, liquid chromatography–tandem mass spectrometry (LC–MS) and nuclear magnetic resonance (NMR) (He et al., 2021). Colorimetry and NMR are susceptible to TMA interference, and their accuracy and sensitivity are limited. Because of its high sensitivity, good specificity and high quantitative accuracy, LC–MS has become the most recognized method to detect TMAO. However, this method takes a long time to prepare samples and depends on expensive professional equipment, so it is not cost-effective. Therefore, it is necessary to continue to explore new detection methods suitable for clinical promotion in the future. After 2015, keywords in TMAO and CVD research broke out, mainly including keywords related to diet, such as red meat, nutrition, high fat diet and mediterranean diet, and keywords related to CVD prognosis, such as cardiovascular risk, prognostic value, cardiovascular event and outcome. This shows that researchers have observed the relationship between diet, TMAO and CVD and the relationship between TMAO and the prognosis of CVD. In the last 3 years, the keywords with the strongest citation busts are endothelial dysfunction and activation, suggesting that the related mechanism research is becoming a new research hotspot in the field of TMAO and CVD.

## 4 Discussion

**Figure 7:**
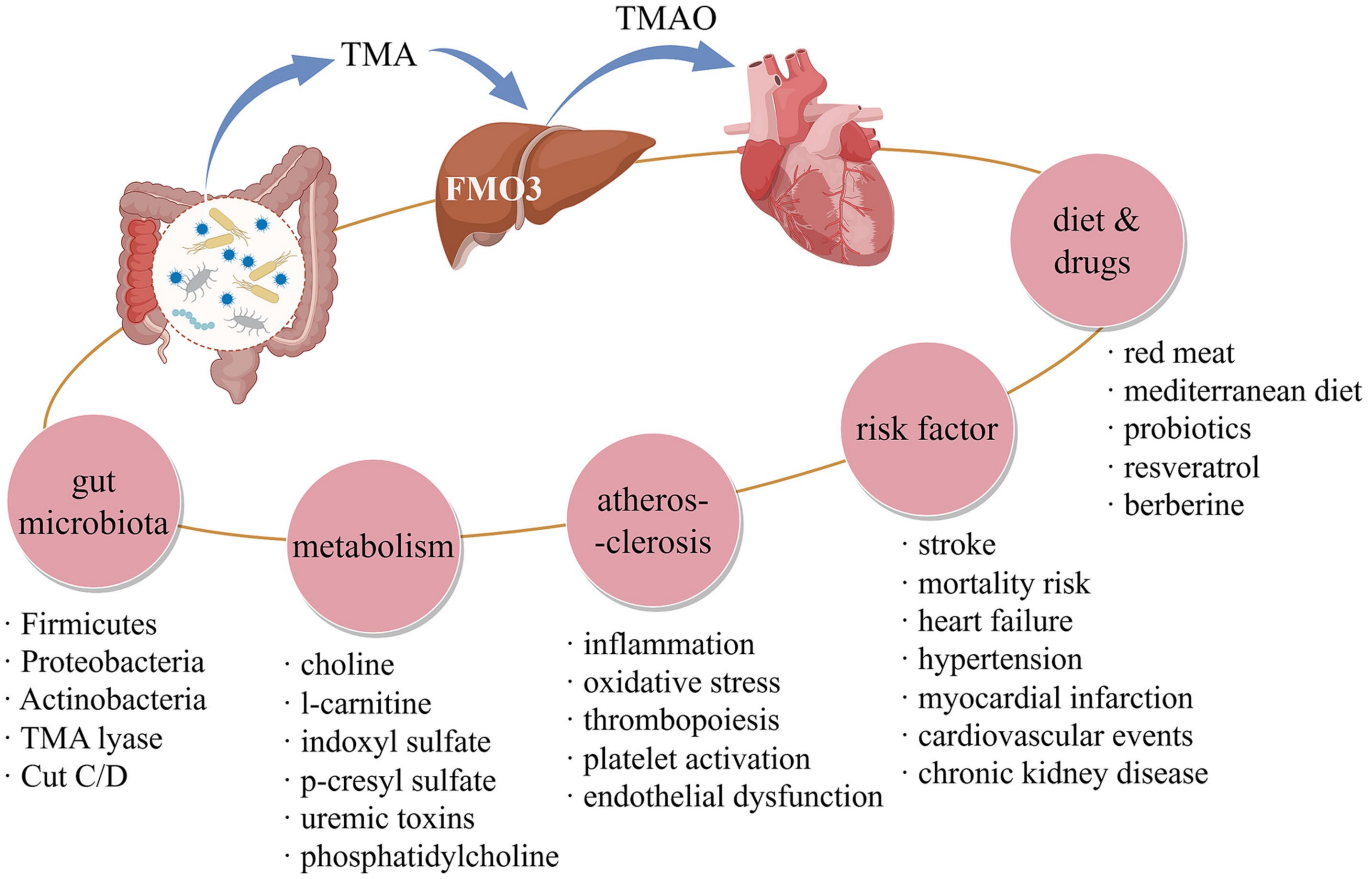
Research focuses and hotspots in TMAO and CVD field. Created by figdraw.

This research methodically compiles and scrutinizes studies exploring the link between TMAO and CVD using bibliometric methods. This study included 1,466 publications from 544 journals, involving 79 countries/regions on five continents, 322 scientific research institutions and 8,135 authors. It can be seen that studies focusing on TMAO and CVD are now widely prevalent globally. Over the last two decades, this domain’s evolution was notably sluggish in its initial decade. However, post-2014, there was a surge in the publication of articles, largely credited to W H Wilson Tang, Zeneng Wang, Robert A Koeth, and Cleveland Clinic colleagues, who authored numerous prominent papers between 2011 and 2014. These works underscored TMAO’s significance in the emergence, progression, and prognosis of CVD, making this field enter the vision of global scholars. Based on the above analysis results of references and keywords related to TMAO and CVD, it is found that the influence of TMAO on the pathological mechanism of AS, the predictive value of TMAO on the prognosis of CVD and the diet and drug intervention for TMAO are the research focuses and hotspots in this field, as shown in Figure 7, which have been discussed later.

### 4.1 TMAO, AS a metabolite of intestinal microbiota, participates in the pathological mechanism of AS

TMAO is derived from TMA produced by the decomposition and metabolism of dietary precursors by intestinal microorganisms. Romano et al. (2015) isolated and cultured 79 common intestinal microorganisms, and found 8 producers of TMA, including *Anaerococcus hydrogenalis, Clostridium asparagiforme, Providencia rettgeri*, etc. The production of TMA involves three microbial-dependent pathways, among which the choline TMA lyase encoded by CutC/D gene and its activating protein pathway and the Rieske oxygenase/reductase pathway encoded by CntA/B gene are the main ones. CutC/D pathway, which takes choline as substrate, is widely found in the strains of *Firmicutes*, *Proteobacteria*, and *Actinobacteria* (Craciun and Balskus, 2012), while CntA/B pathway, which takes carnitine and its derivative *γ* -butyl betaine as substrate, has been found in *Gammaproteobacteria* and *Betaproteobacteria* species (Zhu et al., 2014). Rath et al. (2017) found that CutC/D gene was mainly related to *Clostridium XIVa strains* and *Eubacterium* sp. *strain AB3007*, while CntA/B gene was mainly related to *Escherichia/Shigella* and *Klebsiella* by establishing the database of key genes of TMA synthesis pathway and analyzing the metagenome of fecal samples. A series of molecular mechanisms mediated by intestinal microflora and its metabolite TMAO are closely related to the risk factors and development of CVD (Hemmati et al., 2023). It is reported that the proportion of *Chryseomonas* and *Helicobacter* in Proteobacteria of AS patients is higher than that of healthy people (Karlsson et al., 2012). *Akkermansia muciniphila* has been proved to promote the occurrence and development of AS (Li et al., 2016). In addition, bacterial DNA has been found in AS plaque (Koren et al., 2011). Through the analysis of references and keywords in bibliometrics, it is found that he influence of TMAO on the mechanism of AS is the focus of TMAO and CVD. Moreover, inflammation, endothelial dysfunction, oxidative stress and activation are the keywords related to the mechanism.

AS is a key pathological process in the occurrence and development of CVD, which refers to the accumulation of fat and/or fibrous substances in the intima of arteries (Libby et al., 2019). In 1999, Russell Ross first proposed that AS is a chronic inflammatory disease (Ross, 1999). So far, this view has been widely verified and recognized, and many proinflammatory cytokines, inflammatory mediators and inflammatory signaling pathways have been observed to participate in the occurrence and development of AS (Kong et al., 2022). AS usually starts from the pro-inflammatory activation of vascular endothelium (Gimbrone and García-Cardeña, 2016). Experimental studies have shown that acute injection of TMAO can induce aortic vascular inflammation by activating MAPK and NF-κB signaling cascades in AS-susceptible mice (Seldin et al., 2016). Another study shows that TMAO can partially activate the inflammatory corpuscles of NLRP3 by inhibiting the SIRT3-SOD2-mtROS signaling pathway of apolipoprotein E (ApoE)^−/−^ mice, thus promoting vasculitis (Chen et al., 2017). At the same time, studies have shown that pro-inflammatory microorganisms such as *Bilophila*, *Streptococcus*, and *Mucispirillum* can antagonize the production of SCFAs, aggravate systemic inflammation, and then accelerate the formation of AS (Brandsma et al., 2019). Endothelial dysfunction is the earliest detectable change of AS, which is closely related to endothelial inflammation (Gimbrone and García-Cardeña, 2016). Endothelial dysfunction occurs in the lesion-prone area of arterial vascular system. When this occurs, low-density lipoprotein infiltrates and accumulates into the endothelial layer, at the same time, the cell adhesion molecules is activated, and immune cells migrate to the endothelial layer to induce vasculitis, thus promoting AS (Mazzi et al., 2021). Endothelial dysfunction is characterized by decreased NO production and increased ROS, with inflammation and oxidative stress as important mechanisms for its occurrence and development (Wang and He, 2020). TMAO can regulate the release of proinflammatory cytokines and the activation of adhesion molecules, such as IL-6 and VCAM-1 (Querio et al., 2023), at the same time, it can increase the content of ROS in endothelial cells, reduce the release of NO, and induce oxidative stress in endothelial cells (Zhao and Zhao, 2022). In addition, TMAO can induce endothelial cell inflammation and endothelial dysfunction by activating ROS-TXNIP-NLRP3 inflammasome (Sun et al., 2016). It can be seen that TMAO can activate vascular endothelial inflammation and promote the occurrence and development of endothelial dysfunction in the early stage of AS. In addition, the occurrence of endothelial dysfunction in AS is closely related to the imbalance of intestinal microbiota, and intestinal microbiota is an important target for drug regulation of TMAO to restore endothelial function (Jin et al., 2024; Tian et al., 2023). However, at present, there is still a lack of specific flora-mediated mechanism of TMAO-induced endothelial dysfunction, which needs to be further explored. Platelet activation is the core of inflammatory reaction of AS, and it is also the key step leading to thrombosis of AS (Davì and Patrono, 2007). In 2016, scholars from Cleveland Clinic discovered for the first time that TMAO can induce platelet activation and high reactivity by increasing intracellular Ca^2+^ release, and enhance the possibility of thrombosis in animal models (Zhu et al., 2016). This study also confirmed the regulatory effect of specific intestinal microflora on TMAO level and thrombogenic potential species by constructing aseptic mice and fecal bacteria transplantation animal models (Zhu et al., 2016). For example, *Allobaculum* is positively correlated with high TMAO level and thrombogenic phenotype, while *Candidatus Arthromitus* and *Lachnospiraceae* are correlated with low TMAO level and antithrombotic phenotype (Zhu et al., 2016). In addition, AS is closely related to the activation of immune cells. Pro-inflammatory macrophages are transformed into foam cells and deposited under arterial endothelial cells by phagocytosis of circulating cholesterol and lipoprotein, which leads to AS plaque formation (Eshghjoo et al., 2021). TMAO can increase the risk of AS by increasing the formation of AS-related clearance receptors and foam cells in macrophages (Wang et al., 2011).

To sum up, TMAO can induce AS-related endothelial activation, endothelial dysfunction and thrombosis, and these biological processes are closely related to gut microbiota, inflammatory, oxidative stress and other pathological mechanisms.

### 4.2 Predictive value of TMAO in prognosis of CVD

Through the keywords analysis in bibliometrics, it is found that the influence of TMAO on the prognosis of CVD is another focus. Coronary artery/heart disease, myocardial infarction, heart failure, hypertension, and chronic kidney disease are the diseases that are focused on, while cardiovascular events, mortality risk, outcomes, prognostic value, and risk factors are some related keywords. In 2011, a large clinical cohort study from Cleveland Clinic confirmed that the circulating level of choline, TMAO and betaine, three metabolites of phosphatidylcholine, increased, which can predict the risk of CVD (defined as peripheral artery disease, coronary artery disease, and myocardial infarction) (Wang et al., 2011). This study affirmed the predictive value of TMAO in CVD risk, and attracted extensive attention of scholars in this field. The incidence of major adverse cardiac events (MACE), including death, myocardial infarction, and stroke, is commonly used as an indicator to assess CVD outcomes (Sattar et al., 2021). In 2013, a large-scale clinical study in Cleveland Clinic followed up 4,007 patients who underwent elective coronary angiography for 3 years, and found for the first time that the elevated plasma level of fasting TMAO was an important predictor of the risk of MACE (Tang et al., 2013). In addition, another study by the institution indicates that when the plasma levels of choline and betaine are elevated in patients with CVD, the risk of experiencing MACE increases by 1.9 times and 1.4 times, respectively (Wang et al., 2014). This prediction function is only effective when TMAO increases, but this study did not explore the relationship between TMAO and MACE (Wang et al., 2014). Other studies have shown that the increased concentration of TMAO and its precursors is related to the increased risk of MACE and all-cause death, but has nothing to do with traditional risk factors, but no quantitative evidence is provided (Heianza et al., 2017). A meta-analysis involving 11 prospective cohort studies found that a high circulating TMAO level was significantly correlated with an increase in cardiovascular events risk by 23% and all-cause mortality risk by 55%, which provided quantitative evidence for the association between TMAO and cardiovascular events risk (Qi et al., 2018). Another meta-analysis showed that the relative risk of all-cause mortality in CVD population would increase by 7.6% for every 10 μmol/L increase in plasma level of TMAO, which confirmed the dose-dependent positive correlation between TMAO and CVD mortality for the first time (Schiattarella et al., 2017). It also confirmed that plasma TMAO levels can predict the risk of MACE in patients with acute coronary syndrome in the short term (30 days and 6 months), as well as predict their long-term (7 years) mortality (Li et al., 2017). It can be concluded that the increased plasma level of TMAO can predict the risk of MACE and all-cause death in CVD.

HF is the terminal stage of various CVD, and it is also a common cause of disability and death. Based on the intestinal hypothesis of HF, the decrease of cardiac output and blood stasis in systemic circulation lead to the damage of intestinal mucosal barrier and the increase of intestinal permeability, which can promote microbial translocation into blood circulation, thus making intestinal microflora and its metabolites play an important role in mediating or regulating the pathophysiology of HF (Zhang Y. et al., 2021). Most patients with HF have disorders of intestinal flora and metabolites such as SCFAs, LPS and TMAO (Trøseid et al., 2020). Studies have shown that the increase of plasma TMAO level in HF patients is positively correlated with the abundance of *Escherichia/Shigella* (Hayashi et al., 2018). A prospective cohort study showed that compared with non-HF subjects (3.5 μM), the median plasma TMAO in HF patients increased to 5.0 μM, which was significantly correlated with the HF biomarker BNP (Tang et al., 2014). In addition, the increase of circulating TMAO level can independently predict the higher 5-year mortality risk of HF patients (Tang et al., 2014). Another study showed that the high level of TMAO in patients with chronic HF was positively correlated with NYHA grade, ischemia and poor outcome (Trøseid et al., 2015). Among them, ischemic HF patients have the highest level of TMAO, followed by patients with stable coronary heart disease and patients with non-ischemic HF (Trøseid et al., 2015). A study on chronic systolic HF patients found that the increase of plasma TMAO level was related to more serious left ventricular diastolic dysfunction, and predicted the bad clinical outcome of 5-year death or transplantation (Tang et al., 2015). Therefore, TMAO is an important prognostic marker for HF patients.

Hypertension is the most common chronic CVD in the world and one of the most important modifiable risk factors (Stanaway et al., 2018). The pathogenesis of hypertension has not been fully clarified. The brain-gut axis hypothesis of hypertension holds that the bidirectional communication between intestinal microflora and intestinal epithelial cells in the brain-gut axis participates in the regulation of neuroinflammation and autonomic nervous system, thus raising hypertension (Richards et al., 2022). The diversity and richness of intestinal flora decreased in both hypertensive patients and animal models, and the structure and metabolites of intestinal flora changed (Yang et al., 2015). The abundance of some specific bacteria is positively correlated with the blood pressure level, For example *Parabacteroides*, *Klebsiella*, and *Prevotella* (Verhaar et al., 2020), and some studies have confirmed the therapeutic effects of fecal bacteria transplantation, probiotics and antibiotics on animal models of hypertension. A Mendel randomized study confirmed the causal relationship between TMAO and its precursors and blood pressure (Wang H. et al., 2022). TMAO can promote the pathogenesis of hypertension by activating PERK pathway, interfering with cholesterol transport and activating scavenger receptor (Mutengo et al., 2023). A meta-analysis showed that there was a positive dose-dependent relationship between the level of circulating TMAO and the risk of hypertension, that is, the relative risk of hypertension increases by 9% for every 5 μmol/L increase of circulating TMAO concentration, and by 20% for every 10 μmol/L increase (Ge et al., 2020). Another study shows that TMAO-related aortic stiffness may be related to the increase of blood pressure (Pierce et al., 2021). TMAO may be a potential intervention target in the treatment of hypertension (Zhang et al., 2021).

CKD is a progressive disease with high morbidity and mortality, and CVD is the main cause of death (Matsushita et al., 2022). The pathogenesis of CKD involves kidney-gut axis hypothesis, which holds that the increase of serum urea level induces intestinal flora disorder, and the derived toxins destroy the intestinal mucosal barrier, thus accelerating renal failure, which in turn will further lead to waste retention, aggravate intestinal flora disorder, and lead to excessive production of intestinal-derived uremic toxins such as TMAO, indoxyl sulfate and p-cresyl sulfate and enter the circulatory system, forming a vicious circle (Hobby et al., 2019). Studies have shown that *Actinomycetes, Firmicutes and Proteobacteria* in patients with end-stage CKD have greatly increased compared with healthy people (Vaziri et al., 2013), and these bacteria are all important producers of TMA. Li et al. (2018) showed that the circulating TMAO concentration in CKD rats increased significantly, which was related to vascular oxidative stress, endothelial dysfunction and increased expression of pro-inflammatory cytokines. A study has shown that a high level of plasma TMAO in CKD patients indicates a poor long-term survival rate (Tang et al., 2015). An observational study shows that elevated TMAO level is independent predictors of mortality in CKD3-5 patients (Missailidis et al., 2016). In the late stage of diabetes, CKD often occurs. It has been shown that higher fasting plasma level of TMAO in diabetic patients can predict higher MACE and death risk independently of traditional risk factors, renal function and blood glucose control (Tang et al., 2017).

### 4.3 Diet and drug intervention for TMAO

Through references bursts analysis and keywords co-occurrence analysis in bibliometrics, it is found that the diet and drug intervention for TMAO are also the research focus in this field. Diet has an important influence on TMAO and CVD. TMAO mainly comes from the catabolism of animal-derived diets, including red meat, egg yolk, viscera, whole dairy products, super-processed foods, seafood, etc. Evans et al. (2023). Research indicates that fish naturally possess TMAO, enabling them to skip intestinal flora and liver metabolism and directly enter the bloodstream, swiftly elevating circulating TMAO levels, surpassing those found in eggs or beef (Cho et al., 2017). In addition, the content of TMAO in deep-sea fish is high, while the content of TMAO in fish or seafood in shallow water or fresh water is low (Wang Z. et al., 2022). It is worth noting that the precursors of TMA, phosphatidylcholine, l-carnitine and choline, are rich in high-fat diet, which is considered as a changeable risk factor for CVD (Whitehead et al., 2024). Another study shows that people who eat more eggs and beef produce more TMAO and have lower intestinal microbial diversity, among which *Clostridiaceae*, *Lachnospiraceae*, and *Veillonellaceae* of the phylum *Firmicutes* are more abundant, while people who eat more fruits produce less TMAO and *Bacteroidaceae* and *Prevotellaceae* of the phylum *Bacteroides* are more abundant (Cho et al., 2017). Recently, a large prospective cohort study confirmed that eating a large amount of animal fat (including dairy products and eggs) in the diet was proved to be related to an increased risk of CVD death, whereas eating higher vegetable fat was related to a lower CVD death rate (Zhao et al., 2024). Therefore, the guidelines recommend reducing animal fat intake and increasing intake of fresh fruits and vegetables to reduce CVD risk. The mediterranean diet in Crete is similar to the recommended diet. The mediterranean diet is mainly vegetarian, rich in vegetable fats such as olive oil and rapeseed oil (about 40% of the total energy intake), and emphasizes the intake of whole grains, fruits, vegetables and beans, which is considered to prevent CVD in recent years (Spence, 2018). Mediterranean diet is rich in dietary fiber and polyphenols, which can induce the growth of strains producing SCFAs, such as *Bifidobacteria*, *Bacteroides*, and *Faecalibacterium prausnitzii* (Barber et al., 2023). At the same time, it can reduce the level of TMAO in plasma and urine (Lombardo et al., 2022). In particular, different types of food intake are related to the colonization of specific probiotics, such as grain consumption related to *Bifidobacterium*, olive oil consumption related to *Faecalibacterium*, and legumes consumption related to *Coprococcus* (Merra et al., 2020). Based on the above evidence, it seems that diet, gut microbiota, TMAO and CVD are closely related. However, the current research does not fully support this view. Some studies have shown that dietary choline supplementation does not increase the plasma TMAO level (Wilcox et al., 2021). The addition of carnitine to the diet increased the plasma TMAO level, but the relationship between this change and CVD biomarkers has not been observed (Thomas and Fernandez, 2021). Pignanelli et al. (2018) conducted different modes of dietary intervention on CVD patients, and found that it had no significant effect on plasma TMAO level, and there was no significant correlation between carotid plaque load and Mediterranean diet score. Therefore, the direct causal relationship among diet, gut microbiota, TMAO and CVD cannot be determined at present, and more related research is needed to explore.

The intervention effect of diet on TMAO is still controversial, and drug intervention is very necessary. The production of TMAO depends on the production and transformation of its precursor TMA. Wang et al. (2015) found that 3,3-dimethyl-1-butanol (DMB), a choline analog, can non-fatally inhibit the formation of TMA in microorganisms, and inhibit the formation of macrophage foam cells in ApoE^−/−^ mice fed with high choline diet, thus inhibiting the progression of AS. Subsequent studies have shown that DMB can reverse endothelial dysfunction induced by elevated circulating TMAO in aged rats (Li et al., 2017). Roberts et al. (2018) modified DMB to develop an inhibitor targeting CutC/D. By taking CutC/D inhibitor once orally, the plasma TMAO level can be significantly reduced for 3 days, and the platelet hyperreactivity and thrombosis induced by dietary choline supplementation can be reversed (Roberts et al., 2018). This study provides the possibility of targeting intestinal microbial enzyme/TMA/TMAO pathway to inhibit thrombosis. However, the toxicological evaluation of related drugs needs to be further improved. Through bibliometric analysis, the hot spots of drug intervention mainly focus on probiotics and natural products. The formation of TMAO is dependent on intestinal microbiota. Studies have shown that broad-spectrum antibiotics can inhibit the increase of TMAO level mediated by dietary choline (Tang et al., 2014) and inhibit the occurrence and development of AS (Wang et al., 2011). However, the use of antibiotics has multiple potential hazards to health. In contrast, probiotics may be a safer choice. Fang et al. (2019) found that the administration of *Lactobacillus plantarum* ZDY04 improved the formation of AS plaque in ApoE^−/−^ mice induced by high-fat diet by inhibiting inflammation and oxidative stress, accompanied by the decrease of circulating TMAO level and the activation of NF-κB signaling pathway. The administration of *Enterobacter aerogenes* ZDY01 can significantly reduce the levels of cecum TMA and serum TMAO in choline-fed mice, accompanied by the increase of the abundance of *Bacteroidales family* S24-7 and the decrease of the abundance of *Helicobacteraceae* and *Prevotellaceae* in the intestine (Qiu et al., 2017). This suggests that probiotic preparation may reduce the production of TMA in intestine by regulating microbiota, and then reduce the level of circulating TMAO. Wang Q. et al. (2022) have shown that supplementation of *Bifidobacterium breve* and *Bifidobacterium longum* can significantly reduce *Ruminococcaceae UCG-009* and *Ruminococcaceae UCG-010* of the phylum *Firmicutes*, and inhibit the production of plasma TMAO induced by choline. *Lactobacillus plantarum* ZDY04 was screened by administering a variety of probiotics to mice supplemented with choline diet, which can reduce the levels of cecum TMA and serum TMAO by regulating the abundance of *Lachnospiraceae, Erysipelotrichaceae, Bacteroidaceae and Mucispirillum*, and can significantly inhibit the progress of AS in mice (Qiu et al., 2018). Although the research on the prevention and treatment of CVD by probiotics through TMAO is still limited, several existing studies have shown the potential of probiotic preparations to prevent and treat CVD by regulating gut microbiota to inhibit TMAO.

In recent years, natural products have shown pharmacological advantages in regulating TMAO and preventing CVD because of their natural and safe characteristics. Chen et al. (2016) shows that resveratrol, a natural polyphenol extracted from wild chrysanthemum, berries and other plants, can regulate bile acid metabolism and TMAO biosynthesis by increasing the abundance of *Lactobacillus* and *Bifidobacterium*, thus reducing AS. However, in a recent randomized controlled clinical trial, when resveratrol combined with exercise training intervened in CVD high-risk patients, the supplementation of resveratrol did not significantly affect circulating TMAO while improving other CVD metabolic markers (Baptista et al., 2024). This shows a contradictory conclusion. Berberine is a natural intestinal microbiota regulator extracted from Chinese herbal medicine Coptidis Rhizoma. Animal experiments show that berberine can interrupt the formation of AS plaque induced by high-fat diet by down-regulating choline-TMA-TMAO pathway (Ma et al., 2022). In addition, in further clinical research, it was found that taking berberine orally can reduce plaque score and TMAO content in feces and plasma, showing its efficacy in regulating TMAO to prevent and treat AS (Ma et al., 2022). Wang et al. (2024) used berberine to intervene choline-angiotensin II hypertensive mouse model, and found that berberine could significantly improve vascular dysfunction induced by TMAO, and down-regulate the abundance of *Firmicutes*, especially those with CutC/D gene expression, such as *Lachnospiraceae_NK4A136_group*, *Clostridia_UCG_014*, and *Ruminococcus*. Further experiments *in vitro* showed that berberine could inhibit TMA biosynthesis by binding and inhibiting the activity of CutC/D (Wang et al., 2024). Puerarin is the main effective component of Chinese herbal medicine Lobed Kudzuvine Root. Li Z. H. et al. (2024) shows that puerarin can reduce the level of plasma TMAO by inhibiting *Prevotella copri* and TMA produced by it, thus showing a protective effect on AS. Pterostilbene is a stilbene compound, which widely exists in blueberries, grapes and Guangxi dragon’s blood. Koh et al. (2019) found that pterostilbene can increase the abundance of *Bacteroides*, decrease FMO3 and TMAO, and show anti-vasculitis activity. At present, there are more and more traditional Chinese medicine preparations, such as Qing-Xue-Xiao-Zhi formula (Li et al., 2021), ZeXieYin formula (Liu et al., 2023) and Shenfu injection (Li L. et al., 2024), which also show the effect of inhibiting TMAO in the prevention and treatment of CVD. However, at present, there are few studies on TMAO as the target of TCM intervention in CVD. Li D. et al. (2024) found that Shexiang Baoxin pill, a famous Chinese patent medicine for treating CVD, can down-regulate the level of TMAO and inhibit the apoptosis and oxidative stress of endothelial cells induced by TMAO, thus improving AS. However, its relationship with intestinal microflora needs to be further clarified.

## 5 Limitation

This bibliometrics analysis only includes English literature and WOS core database, but ignores the contribution of other databases and other languages in the field of TMAO and CVD. In addition, when using bibliometrics tools to analyze citation indicators, because of the time delay of citation indicators, articles published recently are slightly inferior, which may have an impact on the overall results. Of course, bursts analysis may make up for some of the shortcomings in this aspect, thus helping to reflect the overall evolution trend in the field.

## 6 Conclusion

The research outlines the evolution of related studies over the last two decades, utilizing bibliometric methods to examine the link between TMAO and CVD. Through the clustering and burst analysis of references and keywords, it is found that the influence of TMAO on the pathological mechanism of atherosclerosis, the predictive value of TMAO on the prognosis of CVD and the diet and drug intervention for TMAO are the research focuses in the past 20 years, while the endothelial dysfunction related to the pathological mechanism of AS is the recent research focus. In addition, in recent years, the research on TMAO has gradually shifted from quantitative determination to intervention, and in this respect, probiotics and natural products show great pharmacological potential, and more and more related articles are being published, which may be the future research focus in this field.

## Data Availability

The original contributions presented in the study are included in the article/Supplementary material, further inquiries can be directed to the corresponding author.

## Footnotes

^1^ https://data.worldbank.org/indicator/NY.GDP.MKTP.CD?view=map
