# Supplementary material for: Impacts of intestinal microbiota metabolite trimethylamine N-oxide on cardiovascular disease: a bibliometric analysis
Source: Front Microbiol. 2025 Jan 6;15:1491731. doi: 10.3389/fmicb.2024.1491731 (PMC11743947; doi:10.3389/fmicb.2024.1491731)
Supplement: Supplementary file 1 [file Table_1.DOCX]

**Supplementary table 1.** Top 22 references with the strongest citation bursts in TMAO and CVD.

| **No.** | **Reference** | **Journal** | **Author** | **Institution** | **Country** | **Year** |
| --- | --- | --- | --- | --- | --- | --- |
| 1 | Gut flora metabolism of phosphatidylcholine promotes cardiovascular disease | *Nature* | Zeneng Wang | Cleveland Clinic | USA | 2011 |
| 2 | Functional interactions between the gut microbiota and host metabolism | *Nature* | Valentina Tremaroli | Sahlgrenska University Hospital | Sweden | 2012 |
| 3 | Carnitine metabolism to trimethylamine by an unusual Rieske-type oxygenase from human microbiota | *Proceedings of the National Academy of Sciences of the United States of America* | Yijun Zhu | University of Warwick | England | 2014 |
| 4 | γ-Butyrobetaine is a proatherogenic intermediate in gut microbial metabolism of L-carnitine to TMAO | *Cell Metabolism* | Robert A Koeth | Cleveland Clinic | USA | 2014 |
| 5 | The contributory role of gut microbiota in cardiovascular disease | *Journal of Clinical Investigation* | W H Wilson Tang | Cleveland Clinic | USA | 2014 |
| 6 | Measurement of trimethylamine-N-oxide by stable isotope dilution liquid chromatography tandem mass spectrometry | *Analytical Biochemistry* | Zeneng Wang | Cleveland Clinic | USA | 2014 |
| 7 | Flavin containing monooxygenase 3 exerts broad effects on glucose and lipid metabolism and atherosclerosis | *Journal of Lipid Research* | Diana M Shih | University of California Los Angeles | USA | 2015 |
| 8 | Prognostic value of choline and betaine depends on intestinal microbiota-generated metabolite trimethylamine-N-oxide | *European Heart Journal* | Zeneng Wang | Cleveland Clinic | USA | 2014 |
| 9 | Prognostic value of elevated levels of intestinal microbe-generated metabolite trimethylamine-N-oxide in patients with heart failure: refining the gut hypothesis | *Journal of the American College of Cardiology* | W H Wilson Tang | Cleveland Clinic | USA | 2014 |
| 10 | Flavin-containing monooxygenase 3 as a potential player in diabetes-associated atherosclerosis | *Nature Communication* | Ji Miao | Harvard Medical School | USA | 2015 |
| 11 | The TMAO-Generating Enzyme Flavin Monooxygenase 3 Is a Central Regulator of Cholesterol Balance | *Cell Reports* | Manya Warrier | Cleveland Clinic | USA | 2015 |
| 12 | Microbiota-dependent metabolite trimethylamine-N-oxide is associated with disease severity and survival of patients with chronic heart failure | *Journal of Internal Medicine* | Marius Troseid | Oslo University Hospital | Norway | 2015 |
| 13 | Gut microbiota-dependent trimethylamine N-oxide (TMAO) pathway contributes to both development of renal insufficiency and mortality risk in chronic kidney disease | *Circulation Research* | W H Wilson Tang | Cleveland Clinic | USA | 2015 |
| 14 | Intestinal microbiota composition modulates choline bioavailability from diet and accumulation of the proatherogenic metabolite trimethylamine-N-oxide | *Mbio* | Kymberleigh A Romano | University of Wisconsin-Madison | USA | 2015 |
| 15 | TMAO: A small molecule of great expectations | *Nutrition* | Marcin Ufnal | Medical University of Warsaw | Poland | 2015 |
| 16 | Intestinal microbiota-dependent phosphatidylcholine metabolites, diastolic dysfunction, and adverse clinical outcomes in chronic systolic heart failure | *Journal of Cardiac Failure* | W H Wilson Tang | Cleveland Clinic | USA | 2015 |
| 17 | Resveratrol Attenuates Trimethylamine-N-Oxide (TMAO)-Induced Atherosclerosis by Regulating TMAO Synthesis and Bile Acid Metabolism via Remodeling of the Gut Microbiota | *Mbio* | Mingliang Chen | Third Military Medical University | China | 2016 |
| 18 | Serum Trimethylamine-N-Oxide Is Strongly Related to Renal Function and Predicts Outcome in Chronic Kidney Disease | *Plos one* | Catharina Missailidis | Karolinska University Hospital | Sweden | 2016 |
| 19 | Increased Trimethylamine N-Oxide Portends High Mortality Risk Independent of Glycemic Control in Patients with Type 2 Diabetes Mellitus | *Clinical Chemistry* | W H Wilson Tang | Cleveland Clinic | USA | 2017 |
| 20 | Trimethylamine-N-oxide (TMAO) response to animal source foods varies among healthy young men and is influenced by their gut microbiota composition: A randomized controlled trial | *Molecular Nutrition & Food Research* | Clara E Cho | Cornell University | USA | 2017 |
| 21 | Gut Microbe-Generated Trimethylamine N-Oxide From Dietary Choline Is Prothrombotic in Subjects | *Circulation* | Weifei Zhu | Cleveland Clinic | USA | 2017 |
| 22 | Circulating trimethylamine N-oxide and the risk of cardiovascular diseases: a systematic review and meta-analysis of 11 prospective cohort studies | *Journal of Cellular and Molecular Medicine* | Jiaqian Qi | Soochow University | China | 2018 |
